# Preoperative biopsies as predictor for the necessity of inguinal lymph node surgery in squamous cell carcinoma of the vulva-a retrospective tertiary center analysis

**DOI:** 10.1007/s00432-020-03263-1

**Published:** 2020-06-07

**Authors:** Caroline Pahmeyer, Fabinshy Thangarajah, Dominik Ratiu, Anne Maria Schultheis, Birgid Schömig-Markiefka, Peter Mallmann, Bernd Morgenstern

**Affiliations:** 1 Department of Obstetrics and Gynaecology, Medical Faculty, University Hospital Cologne, Kerpener Straße 34, 50931 Cologne, Germany; 2 Department of Pathology, University Hospital Cologne, Kerpener Straße 62, 50937 Cologne, Germany

**Keywords:** Vulvar cancer, Preoperative biopsies, Inguinal lymph node biopsies, Depth of infiltration

## Abstract

**Purpose:**

Squamous cell carcinoma of the vulva (SQCV) is the fifth common cancer in women. Necessity of inguinal lymph node surgery depends on the depth of stromal invasion, inducing lymph node surgery, if depth of invasion is more than 1 mm. In this study we tested the prediction of stromal infiltration depth by measurements in preoperative biopsies.

**Methods:**

We analyzed whether a different operative strategy in respect to lymph node surgery would have been chosen based on the pre- or postoperative depth of stromal invasion for each patient. Examination of infiltration depth in preoperative biopsies and surgical specimen were compared.

**Results:**

In total 77 patients were included in this study. Of those 89.6% showed different depths of stromal invasion comparing the pre- and postoperative specimen. Within seventeen patients (22.1%) preoperative depth was 1 mm or less and a postoperative depth was > 1 mm.

**Conclusion:**

We pointed, that only in 77.9% of the patients who should have undergo lymph node surgery based on the postoperative depth of infiltration underwent this procedure. Consequentially in 22.1% of the cases a second operation could not be prevented with a preoperative taken biopsy as indicator for the necessity of lymph node surgery.

## Introduction

One of the greatest factors in reducing mortality of vulva cancer is an appropriate treatment of inguinal lymph nodes (Thangarajah et al. 2019). If the inguinal lymph nodes are clinically suspicious, a systematic inguinal lymphadenectomy is recommended. Following guidelines (AWMF-Leitlinie Stand 2015; Koh et al. 2017; Oonk et al. 2017; RCOG 2014),the indication for operative staging of the inguino-femoral lymph nodes in early stage disease depends on the stromal invasion. Infiltration of 1 mm or less is rarely associated with inguinal node metastases, whereas infiltration depth of 1 or more mm should be treated with lymph node surgery to reduce mortality (AWMF-Leitlinie Stand 2015). Depending on further factors this can be performed as sentinel lymph node biopsy (SNB) or systematic inguinofemoral lymphadenectomy. Following German and international guidelines, a diameter of less than 4 cm of the unifocal tumor, no clinical suspicious lymph node, informed patient consent and compliance to close follow-up are eligibility criteria for SNB (AWMF-Leitlinie Stand 2015; Koh et al. 2017; Oonk et al. 2017; RCOG 2014). In addition, only appropriately trained surgeons should perform this procedure (AWMF-Leitlinie Stand 2015). In case of midline tumors a bilateral groin node dissection is recommended.

To reduce the morbidity of a second operation, preoperative biopsies are taken into consideration not only to confirm the diagnosis of vulva cancer but to determine the need for lymph node surgery depending on the depth of infiltration.

Within this investigation, we took a closer look into the reliability of preoperative biopsies and scrutinized its qualification as indicator for the necessity of lymph node surgery in vulva cancer.

## Methods

Patients with histologically proven SQCV treated in the University Hospital Cologne between 2005 and 2019 and with available preoperative punch biopsies were taken into consideration within this study. Patients underwent primarily surgery of the vulva with or without lymph node surgery depending on preoperative diagnostics. Patients with a depth of infiltration ≤ 1 mm in the preoperative biopsy and > 1 mm within the surgical specimen were treated with secondary lymph node surgery. Examination of infiltration depth in biopsies and surgical specimen were compared. A relevant difference between two depths of infiltration was defined in cases with at least 1 mm discrepancy. Patients characteristics were assessed and analyzed. Patients with preoperative VIN status and postoperative diagnosed invasive vulva cancer were included also.

## Results

In total, 77 patients were included into this study. The mean age was 56.4 years within the study cohort, while mean BMI was 27.5 kg/m^2^. Both characteristics were within the expected range (Alkatout et al. 2015; Brinton et al. 2017).

The majority of patients showed pT1b tumors (70.1%, *n* = 54) and were nodal negative (76.6% *n* = 59). In total, 76.6% of the patients had G2 tumors. Table 1 shows an overview of patient’s characteristics. Preoperatively 37.7% (*n* = 29) showed an infiltration depth of ≤ 1 mm, 62.3% (*n* = 48) > 1 mm, respectively. The examination of the postoperative specimen showed an infiltration depth of ≤ 1 mm in 28.6% (*n* = 22) of cases and > 1 mm in 71.4% (*n* = 55), respectively.

**Table 1:** Patient collective

Characteristic	*n*	%
Age (years)		
≤ 30	4	5.2
31–40	9	11.7
41–50	12	15.6
51–60	20	26.0
61–70	16	20.8
71–80	9	11.7
> 80	7	9.1
BMI (kg/m^2^)		
17 ≤ 18.5	1	1.3
18.5 ≤ 25	33	42.9
25 ≤ 30	24	31.2
30 ≤ 35	7	9.1
> 35	12	15.6
T-Status		
pT1a	12	15.6
pT1b	58	75.3
pT2	7	9.1
N-Status		
pN0	59	76.6
pN1	6	7.8
pN2	5	6.5
pNx	7	9.1
M-Status		
M0	63	81.8
Mx	14	18.2
G-Status		
G1	3	3.9
G2	59	76.6
G3	13	16.9
Gx	2	2.6

**Fig. 1:**
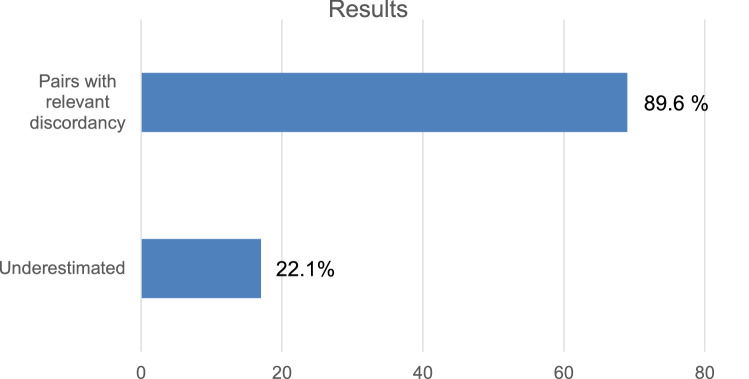
Depth of infiltration/VIN status in preoperative biopsy and operative specimen (underestimated: punch biopsy showed infiltration depth ≤ 1 mm and surgical specimen showed > 1 mm infiltration depth)

Among the study cohort 89.6% (*n* = 69) of the patients showed at least a difference of 1 mm within depths of stromal invasion comparing the pre- and postoperative specimen (Fig. 1). Within seventeen patients the necessity of lymph node biopsies was underestimated- showing infiltration depth ≤ 1 mm within preoperative taken punch biopsy and showing infiltration depth > 1 mm within the surgical specimen. In 22.1% of the cases preoperative biopsies were not able to predict the need for lymph node surgery.

## Discussion

Squamous cell carcinoma of the vulva accounts for 5% of female genital cancers and is currently showing a rising incidence in younger women (Mahner et al. 2015; Thangarajah et al. 2019). One important factor in reducing mortality from vulva cancer is appropriate treatment of inguinal lymph nodes. Sentinel node biopsy was implemented for management of SQCV to reduce morbidity of radical groin dissection such as lymphocele and wound healing complications. It has been proven to be a safe treatment option within early- stage disease (Alkatout et al. 2015; Levenback et al. 2009). Patients undergoing surgery of the vulva for a curative approach of vulva cancer and showing an infiltration depth of > 1 mm should as well be treated with lymph node surgery. Thus, the decision regarding lymph node biopsies in early stage disease directly depends on preoperative the biopsy.

In this study we were able to show that in most patients a difference of at least 1 mm between preoperative infiltration depth and stromal invasion within the surgical specimen was noticed. We pointed out, that only in 77.9% of the patients who should have undergone lymph node surgery based on the postoperative depth of infiltration underwent this necessary procedure. Consequentially in 22.1% of the cases a second operation was required (see Table 2).

**Table 2:** Pre- and postoperative infiltration depth in 77 patients

	Postoperative ≤ 1 mm	Postoperative > 1 mm
Preoperative ≤ 1 mm	15.6% (*n* = 12)	22.1% (*n* = 17)
Preoperative > 1 mm	13.0% (*n* = 10)	49.4% (*n* = 38)

Preoperative infiltration depth: measured in biopsy; postoperative infiltration depth: maximum of infiltration depth in biopsy or surgical tumor specimen

Other studies reviewed whether the depth of infiltration depends on the consulted pathologists and determined the effect of secondary slide reviews of specimen. They showed discrepancies between original and review reports of 0–9%, indicating that slide review should be considered when the infiltration depth is ≤ 1 mm (Beugeling et al. 2014; Chafe et al. 2000; Chan et al. 1999; Khalifa et al. 2003; Santoso et al. 1998; Selman et al. 1999). It might be another approach to review a slide if the infiltration depth is between 0.8 and 1.2 mm. Nevertheless, it remains questionable whether secondary slide reviews are able to solve the problem of different infiltration depths in the pre- and postoperative specimen. Due to the heterogeneous appearance of the tumor itself a biopsy might not be representative regarding the depth of infiltration. Even with secondary slide reviews patients might still not undergo a required lymph node surgery in their first operation but consequently need to get a second operation.

Other preoperative strategies should be considered to avoid a secondary surgery for lymph node biopsy, not only because of its perioperative risks but also because once the tumor is resected an injection near the tumor is not possible anymore and accuracy of sentinel node procedure might be reduced during a secondary operation. Oncological safety of secondary lymph node surgery remains unclear. It should be the aim of modern disease therapy to develop an optimized, individual patient-related strategy and to reduce morbidity. Prospective studies are necessary to evaluate the liability of preoperative biopsies. Taking bigger preoperative biopsies or multiple biopsies might improof accuracy determining the definite depth of infiltration. It remains elusive whether the size of the tumor should dictate the amount or the size of the biopsies.

## Conclusion

Patients who undergo just tumor resection and no primary inguinal lymph node surgery, based on a preoperative depth of infiltration ≤ 1 mm, should be informed about the risk of a secondary operation for lymph node resection. Based on our study, there is a 22.1% risk for the need of a secondary surgery. Studies with a larger group of patients should be performed to further explore this issue.
